# Survivability of *Lactobacillus plantarum* in nutmeg (*Myristica fragrans* Houtt) flesh extract and its effect on the performance of broiler chicken

**DOI:** 10.5455/javar.2023.j650

**Published:** 2023-03-31

**Authors:** Yusri Sapsuha, Said Hasan, Amran Nur

**Affiliations:** 1 Department of Animal Science, Faculty of Agriculture, Universitas Khairun, Ternate, Indonesia; 2 Department of Biology, Faculty of Teacher Training and Education, Universitas Khairun, Ternate, Indonesia; 3 Department of Pharmacology, Biomedicine, Medical Faculty, Universitas Khairun, Ternate, Indonesia

**Keywords:** Broiler, *Lactobacillus plantarum*, nutmeg flesh, performance, synbiotic

## Abstract

**Objective:**

This study aimed to see if increasing the concentration of nutmeg flesh extract *in vitro* could increase the growth of *Lactobacillus plantarum* bacteria and if it had any effect on broiler chicken performance.

**Materials and Methods:**

Different concentrations of nutmeg flesh extract (5, 10, 15, and 20/100 ml distilled water) were combined with 10 ml *L. plantarum* (bacterial concentration 1 × 10^9^ cfu/ml) to produce synbiotics. A total of 250 unsexed Lohmann broiler chickens were reared together from 0 to 7 days of age in the *in vivo* study. Beginning on day 8, synbiotics nutmeg flesh extract and *L. plantarum* were added to the ration in amounts of 0.5, 1, 1.5, and 2 ml/kg for T1, T2, T3, and T4, respectively, while no synbiotics were added to the control diet (T0).

**Results:**

The levels of nutmeg flesh extract had a significant (*p* < 0.05) effect on *L. plantarum* growth. In the survival test against gastric acid, bile salts, and temperature, the addition of nutmeg flesh extract (20/100 ml distilled water) significantly (*p* < 0.05) maintained the population of *L. plantarum*. *In vivo* studies showed that the T1,T2,T3, and T4 groups gained more body weight (*p* < 0.05) than the T0 group during the rearing period but had no effect (*p* > 0.05) on the internal organ weight and carcass of broiler chickens.

**Conclusions:**

Nutmeg flesh extract could stimulate the growth of L. plantarum bacteria, and using it as a synbiotic could improve broiler chicken performance.

## Introduction

The routine use of antibiotic growth promoters (AGP) in broiler feed can cause serious problems for consumers. This is because using AGP can result in residues in chicken products that are harmful to human health [1]. Almost every country in the world has banned the use of AGP in animal feed, including Indonesia, which has done so since 2018. Because AGP prohibition can have a negative impact on broiler chicken productivity and health, an alternative to AGP is required.

Natural ingredient additives, including herbal extract, are replacing antibiotics. Recently, herbal extracts have become popular as natural additives in poultry feed. Among these herbs is nutmeg flesh extract (*Myristica fragrans* Houtt). Because it does not increase production costs, the use of nutmeg flesh extract as an alternative to AGP as a waste from the nutmeg harvest (after taking the main products in the form of seeds and mace) is expected to support the sustainability of broiler chicken cultivation. According to a recent study, using nutmeg flesh extract can improve broiler chicken performance [2]. Nutmeg flesh contains oligo- and polysaccharides, which can act as prebiotics in addition to phytogenic ingredients [3]. According to Gilchrist et al. [4], almost every oligo- and polysaccharide is prebiotic. However, the effectiveness of nutmeg flesh extract as a prebiotic has yet to be established.

Probiotics and herbal ingredients are active ingredients that can be used in place of AGP [1]. Probiotics are live microorganisms that help to maintain the digestive tract’s microflora balance, increase enzyme secretion, vitamin or antimicrobial production, and the host body’s immune system [5]. One type of probiotic that can be used is *Lactobacillus plantarum*. According to Peng et al. [6], using *L. plantarum* B1 improved broiler chicken growth by lowering the content of *Escherichia coli* in the cecum and increasing the content of lactic acid bacteria in the cecum and ileum. Synbiotics are a combination of prebiotics and probiotics that have been shown to improve probiotic survival in the digestive tract [7]. Studies have found that combining prebiotics and probiotics in broiler feed can improve production performance. Compared to a single administration, a combination of prebiotics and probiotics increased body weight and a better feed conversion ratio (FCR) [8,9]. Mohammed et al. [10] and Abdel-Wareth et al. [11] discovered that synbiotic supplementation increased broiler weight gain more than probiotics or prebiotics alone.

Based on the potential of these synbiotics, the use of nutmeg flesh extract combined with *L. plantarum* is expected to be synbiotic that can replace AGP in increasing broiler chicken growth. However, there are no studies in the literature on using nutmeg flesh extract as a synbiotic with *L. plantarum*. This study aimed to see how nutmeg flesh extract concentration affected the growth of *L. plantarum* bacteria *in vitro* and how it affected broiler chicken performance.

## Materials and Methods

### Ethical approval

All procedures performed in the studies involving animals followed the institution’s ethical standards or practices. The research protocol and animal management followed Directive 04/KEPH/PH/2022 of the Animal Ethics Committee, Faculty of Agriculture, Universitas Khairun.

### Production of nutmeg flesh extract

As described by Sapsuha et al. [2], 1 kg of nutmeg flesh flour was extracted by the maceration technique by soaking in a 4 l 96% ethanol solution for 3 × 24 h. During the maceration process, stirring was done twice, once in the morning and once in the evening. The filtrate obtained from the immersion was then filtered and evaporated using a rotary evaporator to produce nutmeg flesh extract.

### Synbiotic production

Synbiotic preparation began by mixing various levels of nutmeg flesh extract (5, 10, 15, and 20/100 ml of distilled water) with 10 ml of *L. plantarum* (bacterial concentration: 1 × 10^9^ cfu/ml). The mixture was then incubated for 24 h at 37°C. The cultures were then planted on Man Ragosa Sharpe *agar* (MRSA) by the pour method and incubated at 37°C for 48 h, and the total population of *L. plantarum* was calculated for testing levels of nutmeg flesh extract.

### Lactobacillus plantarum resistance to gastric acid

The survival of L. plantarum in synbiotic nutmeg flesh extract against gastric acid was tested using a gastric acid solution prepared according to the method of Shehata et al. [12], that is, by mixing pepsin at a concentration of 3 gm/l sterile saline (0.5% w/v) and adding 1 N HCl until the pH reached 2 and filtered through a sterile-filter membrane. Gastric acid fluid was tested by combining 1 ml of synbiotic culture with 9 ml of gastric fluid (pH 2) and incubating it at 37°C for 0 and 2 h. The total colony count was performed after 2 h of incubation to determine the survival of the bacteria in acidic conditions by checking the serial plating in MRSA using the pour method and incubating at 37°C for 48 h. Bacterial survival was calculated using the following formula from Pourjafar et al. [13]:

Bacterial survival = [total final bacterial population (cfu/ml): total initial bacterial population of organisms inoculated with simulated gastric fluid] × 100%.

### Lactobacillus plantarum resistance to bile salts

The survival of *L. plantarum* in synbiotic nutmeg flesh extract was tested against bile salts using the method of Shehata et al. [12], which involved mixing Man Rogosa Sharpe broth (MRSB) with 0.5% bile salt and adding 0.2 N NaOH to pH 8. Samples inoculated with up to 1 ml of gastric fluid were added to 9 ml of bile salts and incubated at 37°C for 0 and 2 h. After 2 h, the total colony count was performed to determine the bacteria’s survival against bile salt conditions using the same method in gastric fluid testing.

### Lactobacillus plantarum resistance to temperature

The temperature resistance of L. plantarum in synbiotic nutmeg flesh extract was tested using the method of Lin et al. [14]. *Lactobacillus plantarum* was heat tested for 15 min in nutmeg flesh extract at 60°C, 70°C, and 80°C. A 1 ml sample was added to 9 ml of MRSB and heated in a water bath for 15 min at the temperature of each treatment. The sample was then planted in MRSA and incubated at 37°C for 48 h. The total number of bacterial colonies was then calculated to determine the bacteria’s resistance to hot conditions.

### In vivo experiment

From 0 to 7 days of age, 250 unsexed Lohmann broiler chickens were reared together. On day 8, broiler chickens (weighing 1882.62 gm) were assigned to one of five treatments, repeated five times. The feed was given as mash and was formulated (Table 1) as starter feed (days 1–21) and finisher feed (days 22–35). From day 8, 0.5, 1, 1.5, and 2 ml/kg rations of synbiotic nutmeg flesh extract and *L. plantarum* were added to the feed for days T1, T2, T3, and T4, respectively. In contrast, the control diet (T0) did not include synbiotic nutmeg flesh extract and *L. plantarum*. Feed and water were available ad libitum until day 35. On day 4, all chickens were vaccinated with the commercial Newcastle disease vaccine via eye drops; on day 18, via drinking water; and on day 12, via water with the Gomboro vaccine. The chickens were reared in a ventilated broiler cage with rice husks as the base. Weekly weight gain, feed consumption, and FCR measurements were taken. On day 35, one chicken was selected randomly from each replicate, slaughtered, and feathered. Internal organs were removed and weighed following evisceration. The percentage of carcasses and the commercial proportion of broiler carcasses were also calculated.

**Table 1.** Feed ingredients and nutrient composition of the research ration.

Items (%, except that otherwise mentioned)	Starter (1–21)	Finisher (22–35)
Yellow corn	56.25	64.10
Fine bran	20.18	15.17
Fish flour	16.73	13.89
Palm oil	2.38	2.38
DL-methionine	0.25	0.25
Bentonite	1.11	1.11
Limestone	1.32	1.32
MCP	1.25	1.25
Premix^a^	0.18	0.18
Chlorine chlorite	0.06	0.06
NaCl	0.29	0.29
Nutrient content based on laboratory analysis:		
ME (kcal/kg)	2,935	3,082
Dry matter	85.62	86.43
Crude protein	21.14	19.05
Extract ether	4.46	4.78
Crude fiber	3.42	3.33
Ash	8.59	9.71

a Premix contained (per kg of diet) of vitamin A 7,750 IU, vitamin D3 1,550 IU, vitamin E 1.88 mg, vitamin B1 1.25 mg, vitamin B2 3.13 mg, vitamin B6 1.88 mg, vitamin B12 0.01 mg, vitamin C 25 mg, folic acid 1.50 mg, Ca-d-pantothenate 7.5 mg, niacin 1.88 mg, biotin 0.13 mg, Co 0.20 mg, Cu 4.35 mg, Fe 54 mg, I 0.45 mg, Mn 130 mg, Zn 86.5 mg, Se 0.25 mg, L-lysine 80 mg, Choline chloride 500 mg, DL-methionine 900 mg, CaCO_3_ 641.5 mg, Dicalcium phosphate 1,500 mg.

### Statistical analysis

The study followed a completely randomized design. The data were analyzed using one-way analysis of variance (SPSS version 16.0), and the Duncan test was used when there was a significant treatment effect (*p* < 0.05). In addition, the influence of additives at different levels in feeds was determined using an orthogonal polynomial contrast test for linear and quadratic effects. A significant impact was considered when *p* < 0.05. When 0.05 ≤ *p* < 0.10 was observed, the tendency was considered.

## RESULTS

### Nutmeg flesh extract level on L. plantarum growth

Table 2 shows how much *L. plantarum* grew when nutmeg flesh extract was added in different amounts. Adding 20/100 ml of nutmeg flesh extract resulted in the greatest growth of *L. plantarum*. After a 24 h incubation period, the level of nutmeg flesh extract significantly (p < 0.05) affected the growth of *L. plantarum*.

### Lactobacillus plantarum resistance to gastric acid

Table 3 displays the survival of *L. plantarum* in synbiotic culture against gastric acid. The results showed that when *L. plantarum* was exposed to acidic conditions and incubated for 2 h in simulated gastric fluid, its viability was significantly (p < 0.05) affected. The initial viability of *L. plantarum* in all synbiotic culture treatments when contacted with acidic conditions in simulated gastric fluid ranged from 9.89 to 10.27 log cfu/ml and after contact with acidic conditions (pH 2) and incubation for 2 h ranged from 7.48 to 9.18 cfu/ml.

### Lactobacillus plantarum resistance to bile salts

Table 4 shows the mean survival data of *L. plantarum* in synbiotic culture against bile salts tested using bile salt simulation. The results showed that adding nutmeg flesh extract significantly (*p* < 0.05) increased the growth of *L. plantarum* at 0 h under bile salt conditions, but only adding nutmeg flesh extract as much as 20/100 ml significantly (*p* < 0.05) increased the growth of *L. plantarum* after 2 h of incubation.

**Table 2.** Synbiotic test on the total population of *L. plantarum* at different nutmeg flesh extract levels.

Items	Nutmeg flesh extract level (ml/100 ml)				SEM	*p*-value		
	5	10	15	20		A	L	Q
Total population of *L. plantarum* (log cfu/ml)	9.98^a^	10.02^b^	10.05^b^	10.18^c^	1.72	<0.01	0.06	0.02

A = Analysis of variance; L = Orthogonal polynomial contrasts test for linear effects, Q = Orthogonal polynomial contrasts test for quadratic effects, SEM = Standard error of the means.

^a,b,c ^Means in the same row with different superscripts differ significantly (*p* < 0.05).

**Table 3.** Synbiotic test on survival of *L.* *plantarum* exposed by stomach acid simulation.

Items	Nutmeg flesh extract level (ml/100 ml)				SEM	*p*-value		
	5	10	15	20		A	L	Q
Exposed to gastric acid for 0 h (log cfu/ml)	9.89^a^	10.02^ab^	10.08^b^	10.27^c^	1.72	<0.01	0.02	0.01
Exposed to gastric acid for 2 h (log cfu/ml)	7.48^a^	8.27^b^	8.21^b^	9.18^c^	0.89	<0.01	0.06	0.07
Survival rate (%)	75.64^a^	82.54^b^	81.46^b^	89.4^c^	0.79	<0.01	0.08	0.08

A = Analysis of variance; L = Orthogonal polynomial contrasts test for linear effects, Q = Orthogonal polynomial contrasts test for quadratic effects, SEM = Standard error of the means.

^a,b,c ^Means in the same row with different superscripts differ significantly (*p* < 0.05).

### Lactobacillus plantarum resistance to temperature

The total population of *L. plantarum* in synbiotic culture was tested for temperature effects at 60°C, 70°C, and 80°C (Table 5). The findings revealed a significant interaction (*p* < 0.05) between nutmeg flesh extract levels and temperature in the population of *L. plantarum*. At the same level of nutmeg flesh extract with increasing temperature, the total population of *L. plantarum* has decreased. In contrast, an increasing level of nutmeg flesh extract at the same temperature level indicates that the total population of *L. plantarum* has increased.

The highest *L. plantarum* bacterial population was found in the 20/100 ml (T4) nutmeg flesh extract level treatment at 60°C. In contrast, the lowest *L. plantarum* bacterial population was found in the 5/100 ml nutmeg flesh extract level treatment at 80°C. These findings show that *L. plantarum* is still heat resistant up to 80°C with the addition of nutmeg flesh extract at levels of 5, 10, 15, and 20/100 ml. The addition of 20/100 ml of nutmeg flesh extract significantly (*p* < 0.05) aided in the maintenance of the *L. plantarum* population.

### Effect of synbiotics on broiler chicken performance

Table 6 shows the performance of broiler chickens fed synbiotic nutmeg flesh extract and *L. plantarum*. During the rearing period, higher body weight gain (*p* < 0.05) was observed in groups T1, T2, T3, and T4 compared to T0, while there were no significant differences (*p* > 0.05) observed between T1, T2, T3, and T4 treatments. Regarding feed consumption, it was discovered that administering synbiotic nutmeg flesh extract, and *L. plantarum* could increase feed consumption (*p* < 0.05) compared to T0. In contrast, there was no significant difference (*p* > 0.05) between T2, T3, and T4 treatments. When compared to T0, T1, and T2, administration of synbiotic nutmeg flesh extract and *L. plantarum* at a rate of 2 ml/kg ration improved feed conversion (*p* < 0.05), but there was no significant difference (*p* > 0.05) when compared to T3.

### Effect of synbiotics on the internal organs of broiler chickens

The results (Table 7) showed that the administration of synbiotic nutmeg flesh extract and *L. plantarum* to broiler chickens had no effect (p > 0.05) on the internal organ weight of broiler chickens.

### Effect of synbiotics on the percentage of broiler carcass

According to the findings (Table 8), the addition of synbiotic nutmeg flesh extract and *L. plantarum* had no effect (p > 0.05) on the percentage of carcasses and commercial cuts of broiler carcasses.

## Discussion

Adding nutmeg flesh extract can provide a substrate for *L. plantarum* growth. According to Adil and Magray [15], using inulin from gembili tubers as a prebiotic can help probiotics grow by providing specific substrates for the fermentation process. To carry out the fermentation process and survive or compete with pathogenic bacteria in the digestive tract, probiotics require prebiotic substrates, typically derived from plants [16]. Indeed, indigestible oligosaccharides found in plants can stimulate the growth and activity of lactic acid bacteria while also increasing the production of volatile fatty acids [17].

**Table 4.** Synbiotic test on survival of *L. plantarum* exposed by bile salt simulation.

Items	Nutmeg flesh extract level (ml/100 ml)				SEM	*p*-value		
	5	10	15	20		A	L	Q
Exposed to bile salts for 0 h (log cfu/ml)	10.14^ab^	10.02^a^	10.19^b^	10.23^b^	1.67	<0.01	0.69	0.53
Exposed to bile salts for 2 h (log cfu/ml)	8.22^a^	8.51^a^	8.48^a^	9.25^b^	1.80	0.02	0.11	0.06
Survival rate (%)	81.08^a^	84.93^a^	83.22^a^	90.44^b^	0.89	<0.01	0.15	0.12

A = Analysis of variance; L = Orthogonal polynomial contrasts test for linear effects, Q = Orthogonal polynomial contrasts test for quadratic effects, SEM = Standard error of the means. ^a,b ^Means in the same row with different superscripts differ significantly (*p* < 0.05).

**Table 5.** Synbiotic test on survival of *L. plantarum* exposed to temperature.

Items	Nutmeg flesh extract level (ml/100 ml)				SEM	*p*-value		
	5	10	15	20		A	L	Q
Exposed to a temperature of 60°C (log cfu/ml)	9.96^ab^	10.28^cd^	10.20^bcd^	10.31^d^	1.58	<0.01	0.21	0.29
Exposed to a temperature of 70°C (log cfu/ml)	9.92^ab^	9.97^ab^	10.09^bcd^	10.28^dc^	1.92	<0.01	0.03	<0.01
Exposed to 80° (log cfu/ml)	9.73^a^	9.92^ab^	10.01^abc^	10.20^bcd^	0.89	<0.01	<0.01	0.02

A = Analysis of variance; L = Orthogonal polynomial contrasts test for linear effects, Q = Orthogonal polynomial contrasts test for quadratic effects, SEM = Standard error of the means. ^a,b,c,d ^Means in the same row with different superscripts differ significantly (*p* < 0.05).

**Table 6.** Productivity of broiler chickens fed with synbiotic nutmeg flesh extract and *L. plantarum.*

Items	Treatment					SEM	*p*-value		
	T0	T1	T2	T3	T4		A	L	Q
Initial body weight (gm)	209.67	208.33	209.32	210.31	210.16	1.58	0.43	0.29	0.21
Final body weight (gm)	1,659.63^a^	1,798.10^b^	1,814.72^b^	1,856.36^bc^	1,878.62^c^	77.56	<0.01	0.02	0.11
Weight gain (gm)	1,449.96^a^	1,589.77^b^	1,605.40^b^	1,646.05^bc^	1,668.46^c^	72.45	<0.01	0.03	0.12
Feed consumption (gm)	2,377.63^c^	2,458.54^b^	2,495.81^a^	2,497.89^a^	2,498.42^a^	30.36	<0.01	0.06	0.21
Conversion ration	1.64^c^	1.55^b^	1.55^b^	1.52^ab^	1.50^a^	0.05	0.02	0.02	0.10

A = Analysis of variance; L = Orthogonal polynomial contrasts test for linear effects, Q = Orthogonal polynomial contrasts test for quadratic effects, SEM = Standard error of the means. ^a,b,c ^Means in the same row with different superscripts differ significantly (*p* < 0.05).

Adding nutmeg flesh extracts at 20/100 ml provided the best bacterial viability, indicating that adding nutmeg flesh to synbiotics increased protection for probiotics against acidic conditions in the gastrointestinal tract (GIT). This is consistent with Muganga et al. [18], who discovered that combining probiotics with prebiotics, particularly those derived from plants, can protect probiotics from the acidic conditions found in the GIT. The ability of *L. plantarum* to survive in gastric acid conditions is critical for probiotics to colonize the host’s GIT [19]. Plant extracts used as prebiotics can protect probiotics inside the host body and when they encounter physical and chemical barriers in the digestive tract [20]. Probiotics can be safeguarded by supplying a substrate for the fermentation process, which generates adenosine triphosphate (ATP) as an energy source [21].

Adding nutmeg flesh extract protected *L. plantarum* from bile salts, resulting in increased growth of *L. plantarum*. These findings were consistent with those of Mena and Aryana [22], who discovered that a 5% lactose addition provided the best results for the growth of *Lactobacillus* bulgaricus. Adding nutmeg flesh extract can increase energy production in the form of ATP via sugar metabolism, thereby inhibiting the negative effect of bile acids on bacterial survival. Bacterial resistance to bile salts is strongly influenced by bile salt hydrolase and changes in the composition of cell membranes and bacterial cell walls [23].

Adding nutmeg flesh extract protects *L. plantarum* from survival during heat treatment. Pan et al. [24] discovered that *L. plantarum* combined with fructo-oligosaccharides (FOS) and exposed to heat at 65°C for 30 min had the highest viability compared to the control. Pan et al. [24] and Nazzaro et al. [25] discovered that some plant oligosaccharides, such as xylene-oligosaccharides, fructo-oligosaccharides, pectin, and inulin, are sources of carbohydrates that can help Lactobacilus survive under stress conditions. Moreover, Ferrando et al. [26] and Wang et al. [27] discovered that adding raffinose to heat-exposed *L. plantarum* can accelerate the cellular recovery of the bacteria.

**Table 7.** Organ weights of broiler chickens fed with synbiotic nutmeg flesh extract and *L. plantarum.*

Items (% live weight )	Treatment					SEM	p-value		
	T0	T1	T2	T3	T4		A	L	Q
Liver	2.63	2.64	2.71	2.74	2.71	0.37	0.87	0.08	0.16
Heart	0.63	0.65	0.63	0.64	0.64	0.04	0.79	0.76	0.82
Proventriculus	0.61	0.67	0.64	0.64	0.65	0.03	0.79	0.54	0.71
Gizzard	1.70	1.66	1.79	1.80	1.61	0.16	0.35	0.90	0.66
Pancreas	0.43	0.44	0.43	0.48	0.42	0.07	0.45	0.83	1.00
Spleen	0.07	0.09	0.09	0.08	0.08	0.04	0.37	0.76	0.94
Thymus	0.18	0.16	0.17	0.17	0.16	0.03	0.94	0.32	0.39
Bursa of fabricius	0.09	0.08	0.09	0.10	0.10	0.06	0.86	0.13	0.09
Deodenum	0.67	0.75	0.68	0.69	0.74	0.15	0.26	0.57	0.56
Jejenum	1.32	1.31	1.32	1.33	1.33	0.12	0.86	0.13	0.09
Ilium	0.68	0.72	0.64	0.70	0.68	0.23	0.27	0.86	0.90
Cecum	0.84	0.82	0.80	0.82	0.83	0.48	0.54	0.73	0.93
Abdominal fat	1.64	1.65	1.63	1.67	1.62	0.32	0.42	0.79	0.65

A = Analysis of variance; L = Orthogonal polynomial contrasts test for linear effects, Q = Orthogonal polynomial contrasts test for quadratic effects, SEM = Standard error of the means.

**Table 8.** Broiler chicken carcasses treated with synbiotic nutmeg flesh extract and *L. plantarum.*

Items	Treatment					SEM	*p*-value		
	T0	T1	T2	T3	T4		A	L	Q
	(% Live weight)								
Eviscerated carcass	68.64	69.08	68.65	68.82	68.73	1.76	0.87	0.91	0.79
	(% Eviscerated carcass)								
Breast	38.73	38.62	35.52	38.67	38.26	2.85	0.39	0.87	0.10
Upper thigh	14.98	14.96	14.91	14.98	14.87	0.97	0.86	0.23	0.21
Lower thigh	11.98	12.11	12.57	12.48	12.20	1.12	0.60	0.37	0.64
Wing	7.18	7.24	7.32	7.08	7.32	0.67	0.60	0.76	0.75
Back	21.35	21.23	21.12	21.64	21.47	1.87	0.91	0.96	0.75

A = Analysis of variance; L = Orthogonal polynomial contrasts test for linear effects, Q = Orthogonal polynomial contrasts test for quadratic effects, SEM = Standard error of the means.

Several studies have found that supplementing broiler chickens with synbiotics can improve body weight gain and feed efficiency [1,28–30]. Yet, there are no studies in the literature that so far explain the effect of the synbiotics nutmeg flesh extract and *L. plantarum* on broiler chicken body weight gain. Our present study found that supplementing broiler chickens with synbiotics like nutmeg flesh extract and *L. plantarum* can improve body weight gain and feed efficiency. The synbiotic effectiveness of nutmeg flesh extract and *L. plantarum* was likely due to the synergistic action of several phytochemicals found in nutmeg flesh [31–33] as well as the probiotic activity of *L. plantarum*, which can maintain microbial balance, improve digestive function, and increase intestinal absorption in broilers [8]. The latter circumstance affects how effectively and efficiently feed is used, which helps broiler chicks grow more quickly. Furthermore, earlier studies have demonstrated that the flesh of nutmeg has hepatoprotective, antibacterial, antiparasitic, antifungal, and anti-coccidiotic properties that can promote the growth of beneficial bacteria, inactivate pathogenic bacteria and facilitate nutrient metabolism and absorption in the digestive tract, all of which can enhance the growth performance of broiler chickens [2]. Broilers fed nutmeg flesh extract and *L. plantarum* improved feed conversion, demonstrating better feed utilization than the control group (T0). According to a different study, adding synbiotics to broiler chicken feed can increase intestinal digestibility, which in turn boosts the growth of the animals [34].

Administration of the synbiotic *L. plantarum* and nutmeg flesh extract has been demonstrated to increase feed intake, which can therefore increase body weight gain in this study. The results of this research were in line with reports of Fadl et al. [35], who found that adding probiotics and prebiotics made from plants increased feed consumption. Reduced stomach emptying time caused by probiotics in feed might enhance feed consumption [35,36]. Additionally, it was claimed that by giving probiotics to broiler chicks, the microbiota in their digestive tracts became balanced, leading to increased feed consumption [37]. In contrast to this conclusion, some investigations found that administering prebiotics and probiotics [38,39] decreased feed consumption. Olnood et al. [40] found that the addition of symbiotics to the feed for broiler chickens resulted in a decrease in feed consumption. Sugiharto [1] asserts that several variables, including probiotic strain, plant type, gender, and dose amount, may contribute to the diversity in results.

The results for internal organs and broiler carcasses in this investigation were congruent with those of Saiyed et al. [41], who discovered no synbiotic effect on a carcass weight or internal organic weight in broiler chickens. According to Sarangi et al. [42], adding mannanoligosaccharide and the synbiotics L. bulgaricus, *L. plantarum*, *Streptococcus* faecium, *Bifidobacterium* bifidus, and *Saccharomyces* cerevisiae to the ration had no impact on the proportion of internal organs and broiler carcasses. In agreement with this, Astuti et al. [43] discovered that feeding broiler chicks a mixture of fermented brown rice and ginger with *Lactobacillus* casei had no impact on the carcasses or the relative weight of internal organs. This reveals that the synbiotic administration of nutmeg flesh extract and *L. plantarum* has no detrimental impact on the features of broiler chicken carcasses. Synbiotics have been shown to benefit the gut of broilers [1]. Several studies have shown the potential benefits of synbiotics on the intestinal microbial ecosystem and immune functions of chickens [8,16,44]. Therefore, an in-depth study regarding the synbiotic administration of nutmeg flesh extract and *L. plantarum* needs to be carried out to see the benefits of synbiotics on the intestinal microbial ecosystem and immune function of chickens.

## Conclusion

The results show that nutmeg flesh extract can make the *L. plantarum* bacteria grow faster and can be used as a synbiotic to help broiler chickens do better.

## List of abbreviations

MRSA, Man Ragosa Sharpe Agar; MRSB, Man Rogosa Sharpe Broth; °C, Degrees celsius; ATP, Adenosine triphosphate; *L. bulgaricus*, *Lactobacillus bulgaricus*; *L. plantarum*, *Lactobacillus plantarum; S. faecium*, *Streptococcus faecium*; *B. bifidus*, *Bifidobacterium bifidus*; *S. cerevisiae, Saccharomyces cerevisiae*.
